# Development of a New Lateral Flow Assay Based on IBMP-8.1 and IBMP-8.4 Chimeric Antigens to Diagnose Chagas Disease

**DOI:** 10.1155/2020/1803515

**Published:** 2020-08-17

**Authors:** Edimilson D. Silva, Ângelo A. O. Silva, Emily F. Santos, Leonardo M. Leony, Natália E. M. Freitas, Ramona T. Daltro, Antônio G. P. Ferreira, Rafaela L. Diniz, Aline R. Bernardo, Alejandro O. Luquetti, Marco A. Krieger, Paola A. F. Celedon, Pedro A. Viñas, Nilson I. T. Zanchin, Fred L. N. Santos

**Affiliations:** ^1^Immunobiological Technology Institute (Fiocruz/RJ), Rio de Janeiro, Brazil; ^2^Gonçalo Moniz Institute (Fiocruz/BA), Salvador, Brazil; ^3^Center of Studies for Chagas Disease, Federal University of Goiás, Goiânia, Brazil; ^4^Carlos Chagas Institute (Fiocruz/PR), Curitiba, Brazil; ^5^Molecular Biology Institute of Paraná (IBMP), Curitiba, Brazil; ^6^Chagas Disease Program, Neglected Tropical Diseases (NTD), World Health Organization (WHO), Geneva, Switzerland

## Abstract

Despite several available methodologies for Chagas disease (CD) serological screening, the main limitation of chronic CD diagnosis is the lack of effective tools for large-scale screening and point-of-care diagnosis to be used in different CD epidemiological scenarios. Taking into account that developing such a diagnostic tool will significantly improve the ability to identify CD carriers, we aimed at performing a proof-of-concept study (phase I study) to assess the use of these proteins in a point-of-care platform using serum samples from different geographical settings of Brazil and distinct clinical presentations. The diagnostic accuracy study was conducted on a panel of two WHO International Standards (IS) and 14 sera from *T. cruzi*-positive and 16 from *T. cruzi*-negative individuals. The results obtained with the test strips were converted to digital images, allowing quantitative comparison expressed as a relative band intensity ratio (RBI). The diagnostic potential and performance were also determined. Regardless of the geographical origin or clinical presentation, all sera with *T. cruzi* antibodies returned positive both for IBMP-8.1 and IBMP-8.4 chimeric antigens. The area under the ROC curve (AUC) values was 100% for both antigens, demonstrating an outstanding overall diagnostic accuracy (100%). Based on the data, we believe that the lateral flow assays based on these antigens are promising methodologies for screening CD.

## 1. Introduction

American trypanosomiasis or Chagas' disease (CD) is an infectious condition caused by the hemoflagellated protozoan *Trypanosoma cruzi*, which is responsible for the highest parasitic disease burden in 21 Latin American countries [1]. According to estimates, CD causes 7,500 deaths annually in the 5.7 million people infected worldwide. To date, 70 million people are at risk of acquiring the infection [1]. There is a high-burden of undiagnosed and untreated individuals, making CD the most important neglected tropical disease (NTD) in the Americas, mainly among poor and marginalized populations [2]. Migration flows and population shifts have favored the dispersion of *T. cruzi*-infected people from Latin America to nonendemic countries from Europe, North America, and Oceania [3].

*T. cruzi* is usually transmitted through contact with feces/urine of infected blood-sucking triatomine bugs, which harbors the parasite in their gut. Additionally, other routes of infection such as blood transfusion, ingestion of contaminated food or beverages, organ donation, and mother-to-child represent an increasingly significant alternative infection pathway. Initial infection is followed by an acute phase, characterized by a high-grade parasitemia. This phase is usually asymptomatic or might present as an unspecific and nonpathognomonic self-limiting febrile illness [4]. Following the acute phase, lasting up 60-90 days, CD enters a lifelong, asymptomatic, indeterminate phase. Nevertheless, after years or decades, 20-30% of *T. cruzi*-infected individuals progress to an advanced life-threatening, debilitating chronic stage with severe cardiac, digestive (typical enlargement of the esophagus or colon), neurological complications or mixed-alterations [5, 6].

During the chronic phase, due to the increasing anti-*T. cruzi* IgG antibodies titers and scarcity or lack of circulating parasites (which are mainly hidden in the heart and digestive muscle), the laboratory diagnosis relies on the utilization of indirect immunoassays, such as enzyme-linked immunosorbent assay (ELISA), indirect immunofluorescence (IIF), indirect hemagglutination (IHA), and point-of-care tests (POC), such as rapid diagnostic tests (RDTs) or lateral flow assays (LFA), particle agglutination, immunodot, and immunofiltration [7]. Despite several available methodologies, operational and technical issues render an equally irregular serological assays performance, which is attributed to the high *T. cruzi* genetic and phenotypic intraspecific diversity [8], choice of antigens employed to sensitize the solid phase of immunoassays [9], variation in disease prevalence [10, 11], and variable immune responses in *T. cruzi*-infected individuals [12]. Accordingly, the World Health Organization (WHO) advises the parallel use of two different serological tests to CD diagnosis.

Currently, the main limitation of chronic CD diagnosis is the lack of effective tools for large-scale screening and point-of-care diagnosis to be used in different CD epidemiological scenarios. In addition, standard protocols are arduous to implement outside of large urban centers and present several constraints, such as the highly trained personnel requirement, the need for specific equipment, refrigerated storage, and the need for the patient to return to health centers several times. This highlights the fact that developing new diagnostic tools adapted to the needs of affected populations and to the reality of health systems based on primary health care and that are easy-to-use will significantly improve the ability to identify CD carriers [13]. A strategy to overcome these limitations, including the irregular performance of serological assays in different settings, is based on the use of recombinant chimeric antigens. These molecules are composed of repetitive and conserved fragments of amino acid sequences of epitopes from several antigenic proteins of the parasite [14, 15].

Considering the predicaments herein set forth, our group recently expressed and purified four *T. cruzi*-chimeric proteins (IBMP-8.1, -8.2, -8.3, and -8.4) and assessed their diagnostic performance in the serologic diagnostic of CD under ELISA or liquid microarray format assays [15–18] in different endemic settings in several Latin American countries [19] and in their immigrants living in Barcelona/Spain [20]. Additionally, these chimeric proteins have shown a negligible cross-reactivity with both American cutaneous and visceral leishmaniasis, demonstrating their utility in regions where *T. cruzi* and *Leishmania* spp. are coendemic [21]. In all previous studies, we observed that the IBMP-8.1 and IBMP-8.4 antigens yielded the highest performance values among the evaluated antigens. In the present study, we endeavored to perform a proof-of-concept study (phase I study) to assess the use of these proteins in a point-of-care platform using serum samples from different geographical settings of Brazil and distinct clinical presentations.

## 2. Materials and Methods

### 2.1. Ethics

Approval for this research was granted by the Institutional Review Board (IRB) for Human Research at the Gonçalo Moniz Institute (IGM), Oswaldo Cruz Foundation (FIOCRUZ), Salvador, Bahia (BA), Brazil (protocol no. 67809417.0.0000.0040).

### 2.2. IBMP Chimeric Antigen Obtainment

Construction of expression vectors and establishment of purification conditions for the *T. cruzi* chimeric antigens, denominated IBMP-8.1 and IBMP-8.4, were described in previous work by Santos et al. [15]. Expression was carried out in *Escherichia coli* strain BL21-Star (DE3), and the proteins were purified by affinity and ion-exchange chromatography using columns and chromatographers supplied by GE Healthcare. Protein concentration was determined using a fluorometric assay (Qubit, Invitrogen).

### 2.3. Lateral Flow Assay (LFA) Design, Assembly, and Testing

**Figure 1:**
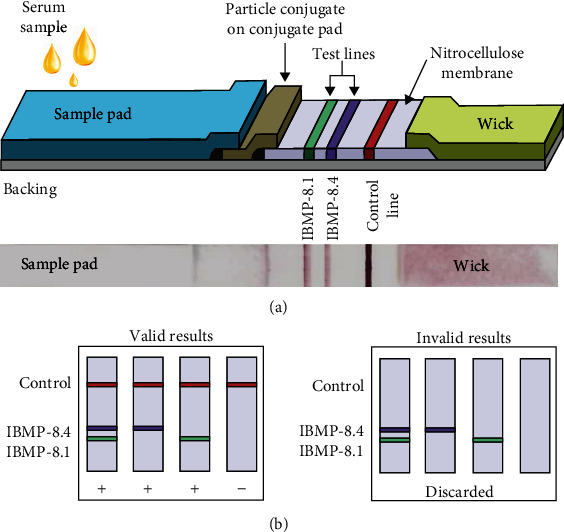
Lateral flow assay (LFA) device. (a) Schematic illustration of the composition of the LFA strip for the detection of IgG anti-*Trypanosoma cruzi* in a clinical sample employing the IBMP-8.4 and IBMP-8.1 chimeric antigens; and photographic image of an LFA opened device after loading *T. cruzi* specific IgG antibodies. (b) Schemes of the results expected for valid and invalid results yielded by LFA.

IBMP-8.1 and IBMP-8.4 *T. cruzi* chimeric proteins were impregnated in parallel with the reaction control on the nitrocellulose membranes used in the lateral flow immunochromatography platform. The test consists of a multimembrane strip sequentially arranged under an adhesive card: sample pad, conjugate pad, antigen and control-impregnated nitrocellulose membrane, and residual membrane or wick (Figure 1(a)). The strip is placed inside a plastic cassette where the reaction takes place. This device has an opening for sample dispensing/running buffer and reaction display window. Five microliters of the sample are dispensed on the opening (Figure 1(a)), followed by the addition of three drops of running buffer to allow the sample liquid to flow through the length of the nitrocellulose membrane. The mixture migrates by capillarity, eluting the conjugate to the test area where the antigens impregnated on the nitrocellulose membrane are found. The overflow of components migrates to the opposite end of the tape where they are retained (wick). Results were obtained visually after 15 minutes of incubation at room temperature (avg. 22°C) or by acquiring a digital photographic image. Positive results are characterized by the presence of color intensity bands in the test (Test line IBMP-8.1 and/or IBMP-8.4) and control (Control line) lines, while negatives show only the control line. If the control line does not appear, the test is considered invalid (Figure 1(b)). In order to avoid variations in the components of assays, the LFA devices were assembled at once (single batch).

### 2.4. Sampling

**Figure 2:**
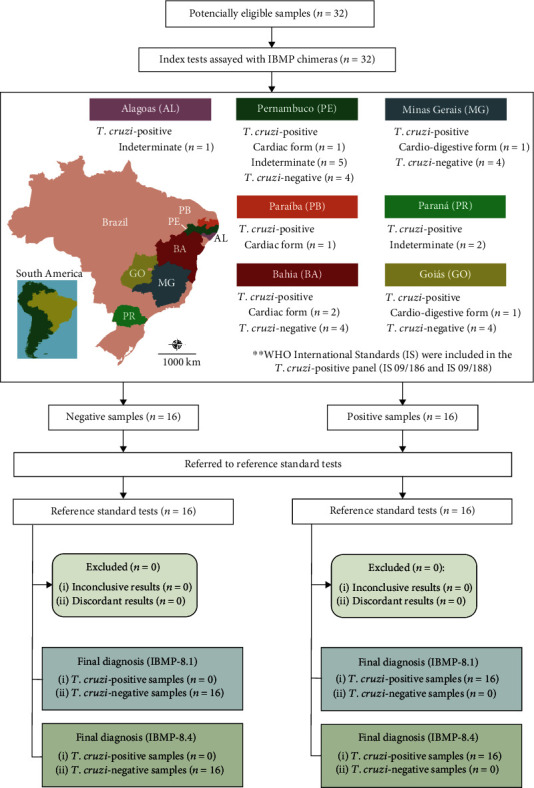
Flowchart illustrating the study design in conformity with the Standards for Reporting of Diagnostic Accuracy Studies (STARD) guidelines. Public domain digital map was freely obtained from the Brazilian Institute of Geography and Statistics (IBGE) cartographic database in shapefile format (.shp), which was subsequently reformatted and analyzed using QGIS version 3.10 (Geographic Information System, Open Source Geospatial Foundation Project. http://qgis.osgeo.org).

Anonymized human serum samples were obtained from the biorepository of the Advanced Public Health Laboratory (LASP/IGM). The sample size was determined for an infinite population (*n* = 1,000,000) with a 95% confidence interval, expected sensitivity and specificity of 99%, and absolute error of 5%. Based on these parameters, obtained with OpenEpi, a free web-based, and open-source program [22], the minimum sample to perform this study was 16 sera from *T. cruzi*-positive and 16 sera from *T. cruzi*-negative individuals. Therefore, 32 serum samples were used in this work. They were obtained from *T. cruzi*-negative (*n* = 16) and *T. cruzi*-positive (*n* = 16) people from Brazilian endemic and nonendemic settings, including Alagoas (AL), Bahia (BA), Goiás (GO), Minas Gerais (MG), Paraiba (PB), Paraná (PR), and Pernambuco (PE) (Figure 2). Additionally, two WHO International Standards (IS) were included in this study: IS 09/186 and IS 09/188, which are representative for seropositive samples from autochthonous individuals living in Brazil, a *T. cruzi* discrete typing unit (DTU) TcII endemic area, and Mexico, a region where *T. cruzi* DTU TcI is endemic. All sera were previously retested for *T. cruzi*-antibodies employing two serological assays: ELISA Chagas III (BIOSChile, Santiago, Chile), which uses whole extracts of Mn and Tulahuen *T. cruzi* strains as antigens, and Gold ELISA Chagas (Rem Diagnóstica, São Paulo, Brazil), which uses both recombinant antigens and purified lysates from Brazilian strains of *T. cruzi* epimastigotes. Additionally, the samples were also retested using an indirect immunofluorescence assay (IFI Chagas, Bio-Manguinhos, Rio de Janeiro, Brazil). All commercial tests were performed under strict adherence to the manufacturer's specifications. Only samples with concordant results were included in the present study. Each sample was given a unique identifier code to ensure a blinded analysis.

### 2.5. Image Analysis

Assays' results interpretation was performed both by visual analysis and captured images of the strip assays at a determinate time of incubation (15 min) by an In Vivo Imaging System (iBox®500 Imaging System, UVP, Upland-CA, USA). Monochrome image data was saved in *.tiff* format, and inverted-color images were obtained using ImageJ [23]. The region of interest was selected on the images where both control and test lines were selected using rectangular selections on the ImageJ menu bar. The same area was applied to all acquired images. Next, areas under the test and control lines peaks were obtained by a virtual chromatogram and quantitatively processed in ImageJ software. In order to normalize the values among readings, the results were expressed as an index that represents the ratio between the test areas and the control area and then divided by the same ratio of the negative control (buffer alone, 0.20 for PBS). This index is referred to as relative band intensity (RBI), corresponding to a ratio towards the negative control value. For quantitative analysis, we have attributed categories to RBI both for IBMP-8.4 and IBMP-8.1 chimeric antigens: P1 (41.0 ≥ RBI ≤ 60.0), P2 (60.0 > RBI ≤ 80.0), and P3 (80.0 > RBI ≤ 100).

### 2.6. Statistical Analysis

Relative band intensities (RBI) were analyzed using scatter computer graphic software (GraphPad Prism version 8, San Diego-CA, USA). Descriptive statistics are presented as geometric means ± standard deviation (SD). The Shapiro-Wilk test was used to test data normality. When the assumed homogeneity was confirmed, Student's *t*-test was used. If not, Mann-Whitney's signed-rank test was used. All analyses were 2-tailed, and a *p* value under 5% (*p* < 0.05) was considered significant. Cut-off values were set by determining the greatest area under the receiver operating characteristic (ROC) curve, which was employed to establish a maximum RBI to distinguish *T. cruzi*-positive and negative samples. Areas under the ROC curve (AUC) were considered to evaluate the global accuracy for each IBMP *T. cruzi*-antigen, which can be classified as low (0.51–0.61), moderate (0.62–0.81), elevated (0.82–0.99), or outstanding (1.0) [24]. LFA performance was assessed using a dichotomous approach and by comparing sensitivity (Se), specificity (Sp), and accuracy (Ac). Confidence intervals at 95% (95% CI) were calculated to address the precision of the proportion estimates of these parameters. Cohen's kappa (*κ*) analysis was employed to determine the agreement strength between the reference standard tests and IBMP-LFA, which was interpreted as a poor agreement (*k* = 0), slight agreement (0.20 ≤ *k* > 0), fair agreement (0.40 ≤ *k* ≥ 0.21), moderate agreement (0.60 ≤ *k* ≥ 0.41), substantial agreement (0.80 ≤ *k* ≥ 0.61), and almost perfect agreement (1.0 ≤ *k* ≥ 0.81) [25]. A checklist (S1 Table) and a flowchart (Figure 2) are provided according to the Standards for the Reporting of Diagnostic accuracy studies (STARD) guidelines.

## 3. Results

**Figure 3:**
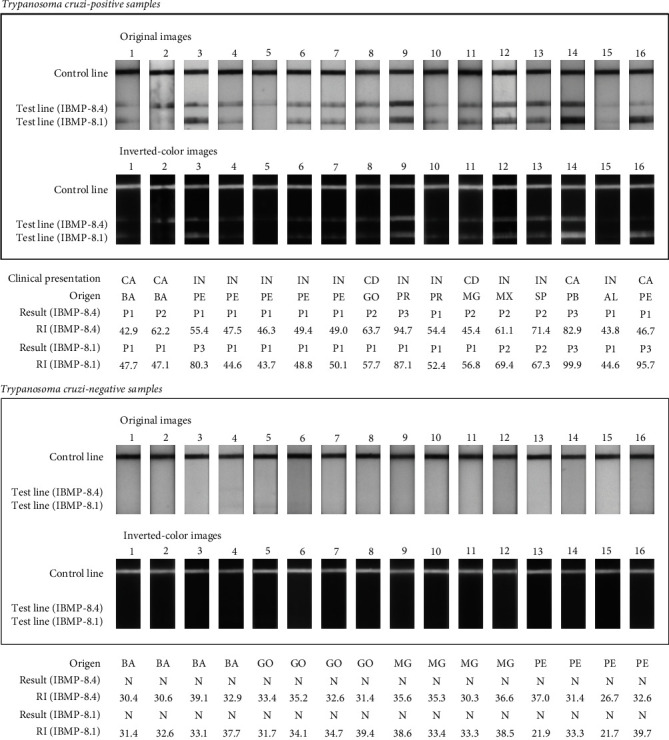
Photographic images of the lateral flow assays (LFA) strips after loading *T. cruzi*-positive (upper panel) or negative (lower panel) samples. BA (Bahia); CA (cardiac form); CD (cardio-digestive form); GO (Goiás); IN (indeterminate form); MG (Minas Gerais); MX (Mexico); N (*T. cruzi*-negative result); P1 (*T. cruzi*-positive result characterized by 41.0 ≥ RBI ≤ 60.0); P2 (*T. cruzi*-positive result characterized by 60.0 > RBI ≤ 80.0); P3 (*T. cruzi*-positive result characterized by 80.0 > RBI ≤ 100); PE (Pernambuco); SP (São Paulo); RBI (relative band intensity).

In order to evaluate the diagnostic performance of the LFA-IBMP, tests were performed using previously characterized *T. cruzi*-positive and negative samples from several CD settings from Brazil. Additionally, the two WHO International Standards (IS) were included in the *T. cruzi*-positive panel (IS 09/186 and IS 09/188). Overall, 32 clinical samples (16 positive and 16 negative samples) were employed in this study. Regardless of the geographical origin or clinical presentation, all sera with *T. cruzi* antibodies returned positive both for IBMP-8.1 and IBMP-8.4 chimeric antigens. Visual analysis of strips showed the presence of bands for all positive samples (Figure 3). Except for samples 5 and 16, which displayed light to nearly fainted colors as a positive indication of detection, all samples presented clear bands for IBMP-8.1, IBMP-8.4, or both. Some samples exhibited whitish stains over the antigen reaction area (samples 1 and 2), which could be related to the production of the devices in a research laboratory without good manufacturing practices. Concerning *T. cruzi*-negative samples no band was observed both for IBMP-8.1 and IBMP-8.4 chimeric antigens. As demonstrated in Figure 3, RBI values found for IBMP-8.1 and IBMP-8.4 antigens varied from 43.7 to 99.9 (geometric mean 59.7; 95% Confidence interval (CI) 51.3-69.7) and from 42.9 to 94.7 (geometric mean 55.7; 95% CI 49.2-63.2), respectively. With respect to negative samples, no false-positive result was observed. All negative samples presented an RBI signal below 40 for both IBMP-8.1 and IBMP-8.4 chimeric antigens. The geometric means of RBI were similar for the two antigens (IBMP-8.1: 34.08, 95% CI 32.36-35.9; IBMP-8.4: 34.04, 95% CI 32.57-35.58). No significant difference has been found with respect to the RBI values considering both *T. cruzi*-positive and negative samples.

**Figure 4:**
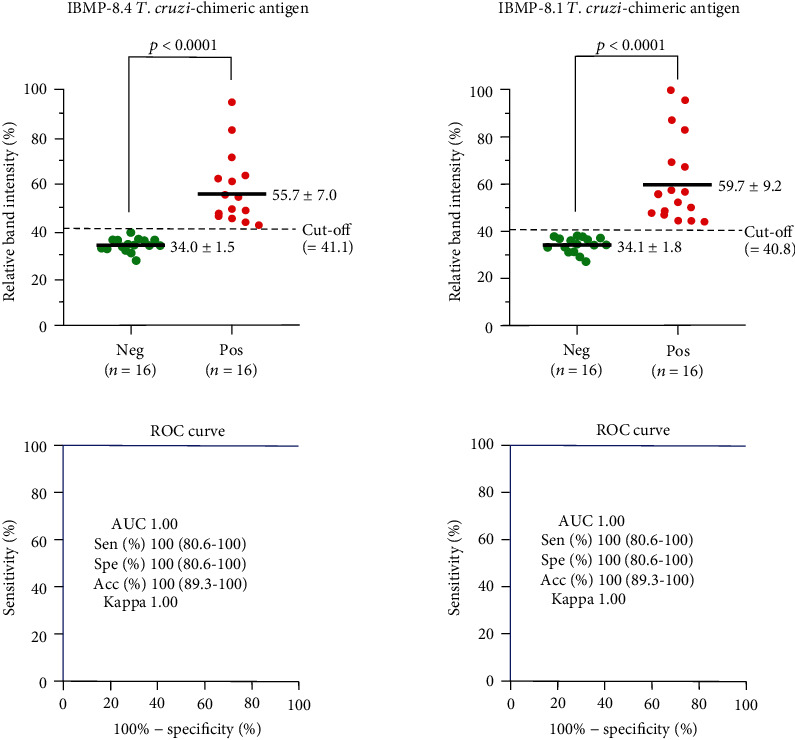
Assay performance parameters were obtained with serum from noninfected and *T. cruzi*-infected individuals. The cut-off value is represented by the dotted lines. Horizontal lines and numbers for each group of results represent the geometric means (±95% CI). Acc (accuracy); AUC (area under curve); ROC curve (receiver operating characteristic curve); Sen (sensitivity); Spe (specificity).

LFA performance parameters found for the IBMP-8.1 and IBMP-8.4 chimeric antigens are illustrated in Figure 4. AUC values reached 100% for both antigens, demonstrating outstanding overall diagnostic accuracy. Cut-off values were established by the ROC curve at 41.1 for IBMP-8.4 and 40.8 for IBMP-8.1. IBMP-8.1 and IBMP-8.4 chimeras yielded maximum values for sensitivity and specificity parameters. Similarly, the accuracy of LFA achieved 100% accuracy for both antigens, diagnosing all individuals correctly. No erroneous results (false-negative or false-positive) were found and no statistically significant differences in sensitivity or specificity scores were found between the chimeric antigens. The qualitative assessment of results showed perfect agreement using the Cohen's Kappa method.

## 4. Discussion

This is the first study using IBMP-8.1 and IBMP-8.4 antigens to detect anti-*T. cruzi* antibodies in a point-of-care device. According to AUC values, these antigens achieved outstanding values of global accuracy (1.0), indicating that they can efficiently distinguish positive from negative samples. In addition, we found a high sensitivity (100%), specificity (100%), and accuracy (100%) values, as well as a perfect agreement between the reference standard tests and IBMP-LFA.

Similar results have already been found for other RDTs employing multiepitope fusion peptides, such as *Trypanosoma* Detect, Simple Chagas WB, and Chagas Detect Plus, as well as recombinant or native antigen matrices, namely, Chagas Stat-Pak, WL Check Chagas, Chagas Quick Test, ICT-Operon, and PATH-Lemos Rapid Test [26–37]. In fact, other studies employing samples from immigrants living in nonendemic settings or from individuals from endemic Latin American countries reported sensitivity between 88% and 100%, while specificity ranged from 94% to 100% [26–37]. In regard to blood donors and *T. cruzi*-positive individuals from Central America, RDT upheld a sensitivity of 99.6% and a specificity reaching 100% [34]. On the other hand, rapid test assessment in Louisiana and Spain revealed high sensitivity (100%) and specificity (between 91.6% and 100%) [35, 37], while a study conducted in Brazil, analyzing samples from different states, namely, Goiás, Minas Gerais, and Bahia, found a sensitivity of 98.5% and specificity of 94.8% [35].

The variation of tests' performances could be a reflection of the high genetic and phenotypic intraspecific diversity of this parasite. Indeed, a peculiar characteristic of this parasite is its high intraspecific genetic and phenotypic plasticity. As a matter of fact, the parasite can exhibit a 48% change in genomic size amongst different strains, a noteworthy characteristic for organisms of the same species [26]. At the current moment, over 6,000 different strains (classified in Discrete Typing Units or DTU's) have been characterized and are geographically heterogeneously dispersed worldwide [38]. Furthermore, this genetic variation has a direct relationship with the parasite antigenic diversity, which is one of the culprit factors for the low concordance between different commercial diagnostic tests employed at different settings. To circumvent the issues associated with elevated antigenic plasticity, chimeric antigens, composed of several conserved, immunodominant, and repetitive epitopes, can be a panacea to address the low agreement between test performance values when employed at different locations with distinct circulating parasite strains [14].

Taking in consideration the lack of agreeability between tests' performance in different areas, our group previously assessed the performance of four *T. cruzi* chimeric antigens in various platforms, such as ELISA and liquid microarray (LUMINEX) [15, 16–18]. To expand our studies, we tested in 32 clinical samples from people of distinct geographical regions in this investigation. In our panel, 20 samples originated from the Brazilian Northeastern states (Alagoas, Pernambuco, Paraíba, and Bahia), where the DTUs TcI and TcII have been identified and associated with the domestic cycle of the disease, while the DTUs TcIII and TcIV, despite being locally present, are associated with sylvatic hosts. Furthermore, we included 12 samples from Brazil's Central-West (Goiás), Southeast (Minas Gerais), and South (Paraná) regions, where the DTUs TcI, TcII, and TcVI are present and associated with the domestic cycle of the disease. Additionally, we also utilized two WHO International Standards samples: one from Mexico, where the DTU TcI is predominant and one from Brazil, where the TcII is predominant. Considering the varying intensity of antigen presentation according to the form and severity of the disease, we decided to utilize samples of patients with the three main chronic presentations of Chagas disease: the Indeterminate (8), cardiac (4), and cardiodigestive (2) forms.

A previous multicenter study evaluated 11 commercially available RDTs for Chagas disease, of which 8 were recommended for *in vitro* diagnostics [7]. However, some technical issues were identified in some of those, such as complex execution procedure (Simple Chagas WB), inconclusive results, and ambiguous manufacturer instructions (Chagas-Instantest and Immu-Sure Chagas), in which could explain the performance variation. In addition, sensitivity and specificity results were different from those reported by the manufacturers. Despite the excellent performance of the ImmunoComb II Chagas Ab kit and Serodia-Chagas, they resemble the ELISA and agglutination tests, respectively [7]. These studies reinforce what has already been discussed by the scientific community in the context of CD diagnosis, such as the urgent need of a simple diagnostic tool with high sensitivity, specificity, speed, and ease of handling. Additionally, the assay should be robust enough to be executed in remote areas without laboratorial infrastructure and have an elevated diagnostic performance for samples from both endemic and nonendemic areas, which would also improve epidemiological surveys. The capacity to detect an early infection and the potential to be used to assess treatment efficiency, thereby increasing the chances of cure and preventing transmission by secondary pathways, would also be a great contribution towards managing Chagas disease [39–41].

Despite a consistent pattern of detection of the control lines, we observed that some LFA exhibited whitish spots over the antigen reaction area, while others displayed light to nearly fainted colors as a positive indication of detection. These effects could have been due to the production of the devices. In fact, LFA devices were not produced in industrial production facilities and machinery using Good Manufacturing Practice (GMP). This is the main limitation of our study. Herein, LFAs used were produced as a prototype to assess the ability of IBMP antigens to differentiate *T. cruzi*-positive samples from those negative. Moreover, different intensities patterns are expected in *T. cruzi*-positive samples, among patients, between proteins (IBMP-8.1 and IBMP-8.4), and also from the same patient collected at different periods. In the future, our group will carry out a phase II study using a high number of samples and LFA devices produced in an industrial laboratory. Despite these limitations, *T. cruzi*-positive samples showed visible reactivity for the IBMP-8.1 and/or IBMP-8.4 antigens, while lack of bands was observed in *T. cruzi*-negative samples, suggesting that they are eligible to enter phase II studies.

## 5. Conclusion

The results described in the present study show a high CD diagnostic capacity of two *T. cruzi* chimeric antigens in lateral flow assays (LFA) using samples with different clinical forms of Chagas disease. These results provide the basis for the development of LFA to be employed as point-of-care assays, presenting fast results either in healthcare facilities or in remote areas with limited resources. However, a large-scale study will be performed in order to validate and confirm the results observed so far.

## Data Availability

The data supporting the conclusions of this article are provided within the article. The datasets generated and analyzed during the current study are available upon request to the corresponding author.

## Supplementary Materials

Supplementary Materials List of Essential Items for Reporting Diagnostic Accuracy Studies (STARD).
